# Relationship Between Venules and Perivascular Spaces in Sporadic Small Vessel Diseases

**DOI:** 10.1161/STROKEAHA.120.029163

**Published:** 2020-04-08

**Authors:** Angela C.C. Jochems, Gordon W. Blair, Michael S. Stringer, Michael J. Thrippleton, Una Clancy, Francesca M. Chappell, Rosalind Brown, Daniela Jaime Garcia, Olivia K.L. Hamilton, Alasdair G. Morgan, Ian Marshall, Kirstie Hetherington, Stewart Wiseman, Tom MacGillivray, Maria C. Valdés-Hernández, Fergus N. Doubal, Joanna M. Wardlaw

**Affiliations:** 1 From the Centre for Clinical Brain Sciences (A.C.C.J., G.W.B., M.S.S., M.J.T., U.C., F.M.C., R.B., D.J.G., O.K.L.H., A.G.M., I.M., K.H., S.W., T.M., M.C.V.-H., F.N.D., J.M.W.), University of Edinburgh, Scotland.; 2 UK Dementia Research Institute (A.C.C.J., G.W.B., M.S.S., M.J.T., U.C., F.M.C., D.J.G., O.K.L.H., S.W., M.C.V.-H., F.N.D., J.M.W.), University of Edinburgh, Scotland.; 3 Centre for Cognitive Ageing and Cognitive Epidemiology (J.M.W.), University of Edinburgh, Scotland.

**Keywords:** brain, humans, risk factors, small vessel disease, venules, venular insufficiency, systemic

## Abstract

Perivascular spaces (PVS) around venules may help drain interstitial fluid from the brain. We examined relationships between suspected venules and PVS visible on brain magnetic resonance imaging.

**Methods—:**

We developed a visual venular quantification method to examine the spatial relationship between venules and PVS. We recruited patients with lacunar stroke or minor nondisabling ischemic stroke and performed brain magnetic resonance imaging and retinal imaging. We quantified venules on gradient echo or susceptibility-weighted imaging and PVS on T2-weighted magnetic resonance imaging in the centrum semiovale and then determined overlap between venules and PVS. We assessed associations between venular count and patient demographic characteristics, vascular risk factors, small vessel disease features, retinal vessels, and venous sinus pulsatility.

**Results—:**

Among 67 patients (69% men, 69.0±9.8 years), only 4.6% (range, 0%–18%) of venules overlapped with PVS. Total venular count increased with total centrum semiovale PVS count in 55 patients after accounting for venule-PVS overlap (β=0.468 [95% CI, 0.187–0.750]) and transverse sinus pulsatility (β=0.547 [95% CI, 0.309–0.786]) and adjusting for age, sex, and systolic blood pressure.

**Conclusions—:**

Despite increases in both visible PVS and suspected venules, we found minimal spatial overlap between them in patients with sporadic small vessel disease, suggesting that most magnetic resonance imaging-visible centrum semiovale PVS are periarteriolar rather than perivenular.

## Abstract 2

Supplemental Digital Content is available in the text.

Perivascular spaces (PVS) are fluid-filled spaces surrounding small perforating brain blood vessels.^1^ They may be part of the glymphatic system^2^ and be important for brain fluid drainage. When enlarged, PVS are visible on T2-weighted and T1-weighted magnetic resonance imaging (MRI) as round or linear hyperintensities/hypointensities respectively, primarily in the basal ganglia and centrum semiovale (CSO). They are neuroimaging features of small vessel disease (SVD).^1,2^ The glymphatic system concept is mostly based on rodent studies: drainage routes, flow mechanisms, and direction of fluid movement are unclear.^2^

Deoxygenated venous blood provides intrinsic contrast on gradient echo and susceptibility-weighted imaging sequences; therefore, vessels visible on these sequences are suspected to be venular. Several visual and computational venular quantification methods have been described (Table IV in the Data Supplement).^3^

We examined spatial relationships between suspected venules and PVS and determined associations between venules and patient demographics, risk factors, SVD features, cerebral microvessel dysfunction, and retinal venules in patients with SVD.

## Methods

We used data from 2 prospective studies of sporadic SVDs: iSVD study (Inflammation in SVD)^4^ and the MSS-3 (Mild Stroke Study 3). Both studies recruited patients with lacunar or minor nondisabling ischemic stroke (modified Rankin Scale score, <3) from NHS Lothian clinical stroke services and used similar 3T MRI sequences (Table V in the Data Supplement). MRI, demographic, and clinical data were obtained within 3 months after stroke. Patients gave written informed consent. The studies were approved by the South East Scotland Research Ethics Committee (references 14/SS/1081 and 18/SS/0044). Data are accessible from the corresponding author.

MSS-3 performs phase-contrast MRI to measure pulsatility in the superior sagittal sinus, straight sinus, and transverse sinuses^5^ and calculates the pulsatility index as (Flow_maximum_−Flow_minimum_)/Flow_mean_.^5^

To quantify suspected venules, we obtained a total venular count in a region of interest in periventricular CSO on gradient echo/susceptibility-weighted imaging. The mean difference in venule-PVS overlap between 2 observers was zero (95% CI, −1 to 1) in interobserver reliability analysis (Data Supplement). One observer performed a total CSO PVS count on T2-weighted images in the venular region of interest. We compared gradient echo/susceptibility-weighted imaging with T2-weighted images to determine definite, probable, or possible overlap of venules and PVS, from their location, shape, and direction (Figure; detailed Methods in the Data Supplement).

**Figure.**
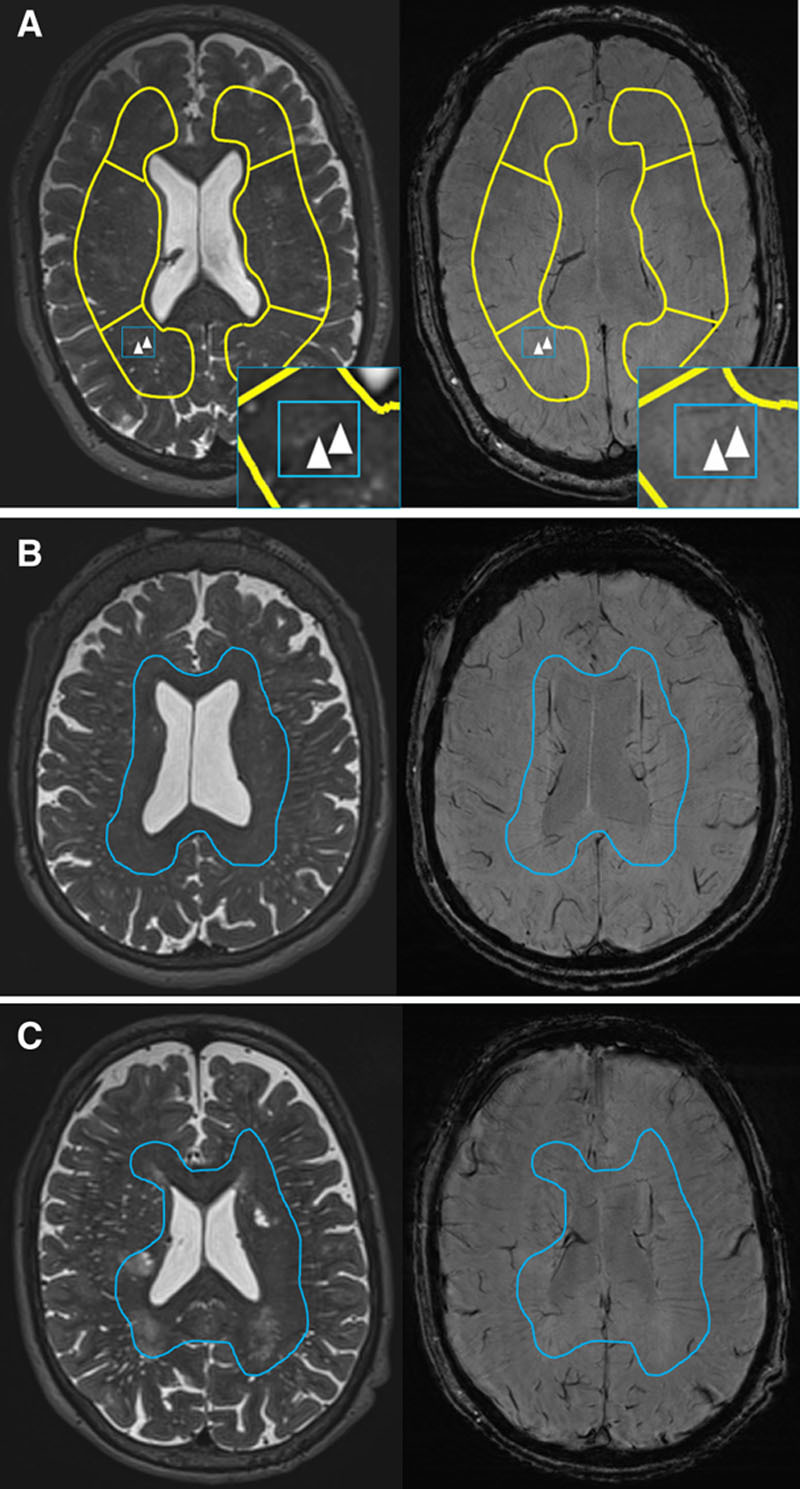
Examples of venules related to perivascular spaces (PVS). **A**, Example of overlap of linear PVS (left inlay, arrowheads) and venule (right inlay, arrowheads). **B** and **C**, Examples of venules (inside blue lines) and PVS (outside blue lines) in different locations.

On retinal images, we measured arteriolar and venular widths (central retinal arteriolar equivalent and central retinal venular equivalent, respectively) and arteriole-to-venule ratio.

We quantified SVD lesions^1^ using Fazekas scale^6^ for periventricular and deep white matter hyperintensities (WMH) and 5-point scale for PVS^7^ (for MRI, retinal imaging, processing, and analyses, see the Data Supplement).

We performed statistical analyses using IBM SPSS, version 24.0 (IBM Corp, Armonk, NY). We used both studies to develop the visual quantification method and assess the venule-PVS spatial relationship (details in the Data Supplement). In MSS-3, we additionally analyzed venular count versus patient demographics, vascular risk factors, SVD features, retinal vessels, and venous sinus pulsatility using multivariable linear regression adjusted for age, sex, and systolic blood pressure. We assessed assumptions of normality with standard methods: no assumptions appeared to be violated. In secondary multivariable analyses, we explored associations between significant predictors from the first analyses in addition to age, sex, and systolic blood pressure. Analyses were explorative, so no formal correction for multiple comparisons was done.

## Results

We included 67 patients (Table 1). Venules were most visible near the ventricles, whereas PVS were most visible adjacent to cortex (Figure [B and C]). When many PVS were present, more PVS were visible near the ventricular outer surface. Even when PVS overlapped with venules, PVS shapes often differed from the venule (Figures IV and V in the Data Supplement).

**Table 1.**
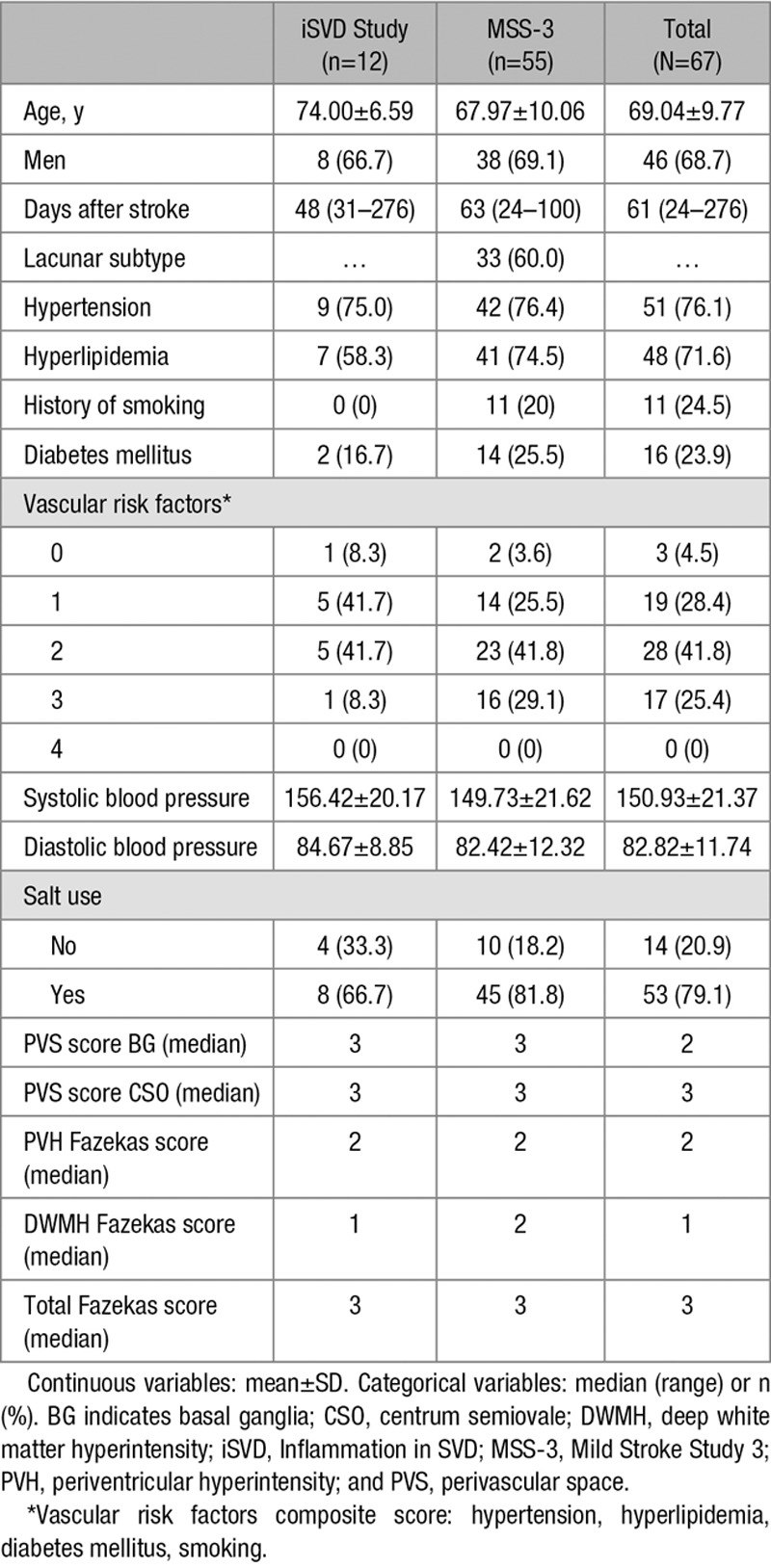
Demographics

Per participant, the mean venular count was 33.22±11.83, mean PVS count was 55.33±28.62, and the median number of venule-PVS overlap was 1 (range, 0–8): only 81 venules had overlapping PVS in all 67 patients (mean percent of total venules that overlapped with PVS, 4.6%; range, 0%–18%; Figure VI in the Data Supplement).

Venular count increased with CSO PVS score (β=0.331 [95% CI, 0.058–0.604]), total CSO PVS count (β=0.605 [95% CI, 0.376–0.835]), and venule-PVS overlap (β=0.500 [95% CI, 0.256–0.744]). Lower venular count was associated with increased pulsatility in the sagittal (β=−0.425 [95% CI, −0.754 to −0.096]) and transverse (β=−0.406 [95% CI, −0.712 to −0.100]) sinuses (Table 2). No other associations were found (Tables VI and VII in the Data Supplement).

**Table 2.**
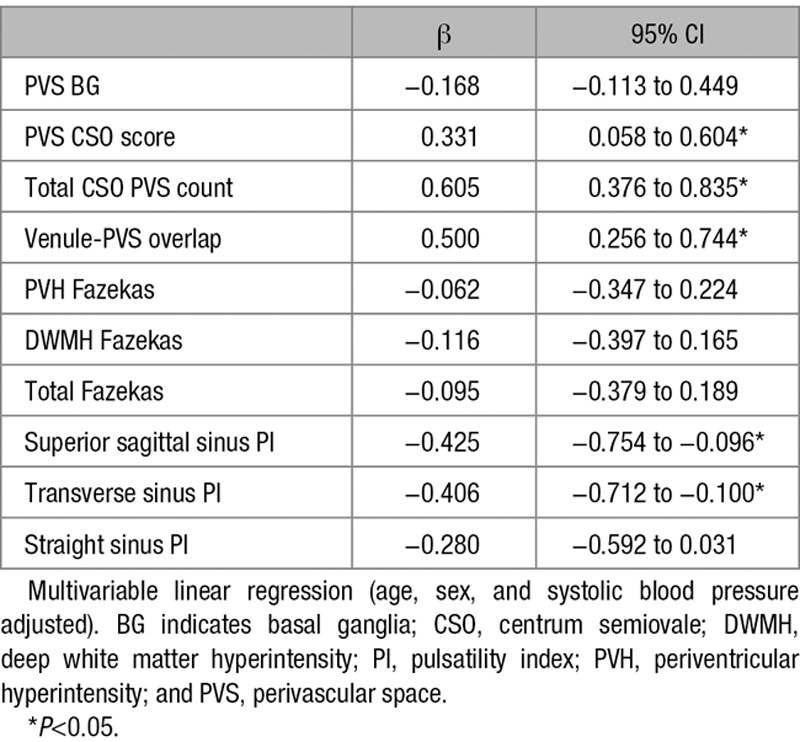
Venular Count Association With Small Vessel Disease Features and Venous Sinus Pulsatility

On substituting total CSO PVS count^8^ for PVS score in the model, venular count remained positively associated with PVS count (β=0.468 [95% CI, 0.187–0.750]) but not venule-PVS overlap. Venular count was still associated with total PVS count (β=0.547 [95% CI, 0.309–0.786]) in the model with transverse sinus pulsatility index.

## Discussion

We found different locations and infrequent overlap between suspected venules and PVS on 3T MRI, suggesting that most venules and MRI-visible PVS are not spatially related. A 7T MRI study^9^ and a pathology study^10^ also found little venule-PVS overlap, suggesting that MRI-visible PVS in humans might be periarteriolar. As PVS increased, punctate PVS (possibly representing PVS around lenticulostriate arterioles from the basal ganglia^10^) more often overlapped with venules at the ventricular edge. Since venular count increased with total CSO PVS count, visible venules and PVS might both indicate worsening SVDs.^2,11,12^

As this may be the first study of venular associations with demographics, risk factors, and MRI measurements, the analyses were exploratory. One study found associations between increased venule visibility and WMH volume,^11^ whereas another found the opposite.^13^ We did not find an association with WMH, perhaps due to the small sample. Contrary to rodent studies,^12^ we did not find associations between venules and hypertension. We also found no associations with retinal vessel widths. Retinal vessel density or fractal dimension may be more sensitive and should be examined in future.

Previous work suggested that PVS increase with increased arterial and venous sinus pulsatility.^14^ We found positive associations between venular count and total CSO PVS count, suggesting that higher venular count might associate with increased pulsatility index but that higher venular count was associated with lower venous sinus pulsatility index. This might be indirectly explained by previous associations found between fewer visible venules and worse WMH^13^ and worse WMH with increased venous sinus pulsatility.^14^

Our study is limited by the small sample and cross-sectional design. Artifacts like vessel calcification might be confused with venules. Strengths include developing an easy-to-apply venular quantification method for gradient echo and susceptibility-weighted imaging sequences. Future studies should examine longitudinal data from larger samples, assess changes over time and more associations.

## Conclusions

Although we did not find a spatial relationship between suspected venules and PVS, more venules seemed to relate to enlarged PVS in the CSO. While we cannot exclude the presence of PVS around venules, enlarged PVS, as visible on MRI, might be more periarteriolar than perivenular.

## Sources of Funding

This study was supported by the Fondation Leducq (16 CVD 05), Row Fogo Charitable Trust (BRO-D.FID3668413), UK Dementia Research Institute (Medical Research Council, Research Councils UK, Alzheimer’s Society, Alzheimer’s Research UK), Wellcome Trust–University of Edinburgh Institutional Strategic Support Fund, SINAPSE (Scottish Imaging Network: A Platform for Scientific Excellence; Scottish Funding Council, Chief Scientist Office), NHS Lothian Research and Development Office (Dr Thrippleton), Stroke Association Princess Margaret Research Development Fellowship (U. Clancy and G.W. Blair), Alzheimer Society (AS-PG-14-033), EU Horizon 2020 (666881, SVDs@Target; G.W. Blair), Garfield Weston Foundation-Stroke Association Senior Clinical Lectureship, NHS Research Scotland (Dr Doubal), Chief Scientist Office Scotland (U. Clancy), Siemens Healthineers (A.G. Morgan), and Alzheimer Nederland (A.C.C. Jochems).

## Disclosures

A.G. Morgan receives funding from Siemens Healthineers. The other authors report no conflicts.

## Supplementary Material


