# Supplementary material for: Relationship Between Venules and Perivascular Spaces in Sporadic Small Vessel Diseases
Source: Stroke. 2020 Apr 8;51(5):1503–6. doi: 10.1161/STROKEAHA.120.029163 (PMC7185057; doi:10.1161/STROKEAHA.120.029163)
Supplement: Supplementary file 1 [file str-51-1503-s001.pdf]

SUPPLEMENTAL MATERIAL

Supplemental methods

Development of ROI

For the visual quantification we initially explored a rectangular region of interest (ROI) of 1 x 3 cm (Figure IA) in periventricular centrum semiovale (CSO) of both hemispheres directly next to the ventricles (see Methods Figure I for overview of the development of ROI). It included the three slices below the uppermost slice where cerebrospinal fluid (CSF) was still visible. With this ROI we would miss out on venules and perivascular spaces (PVS) near anterior and posterior horns of the lateral ventricles.

Subsequently we changed the ROI, now consisting of separate anterior, middle and posterior sections (Figure IB). To start, we applied this ROI in eight patients and counted venules and PVS in all three slices. Venule count, PVS count and overlap showed little difference between the three slices. When lesions or white matter hyperintensities (WMH) were more prominent in one slice than the other slices, we found differences. Because of this little difference, and to decrease the amount of time spent on counting, we chose to continue with a ROI in one slice, located two slices below the uppermost slice where CSF is visible on the gradient echo (GRE) or susceptibility weighted imaging (SWI). The size of the ROI was increased laterally to cover most periventricular WM in the slice to include more venules and PVS and it is divided into anterior, middle and posterior sections (Figure IC and Figure II). For all patients the ROI was manually drawn and adjusted to fit in both T2-weighted and GRE or SWI sequences.

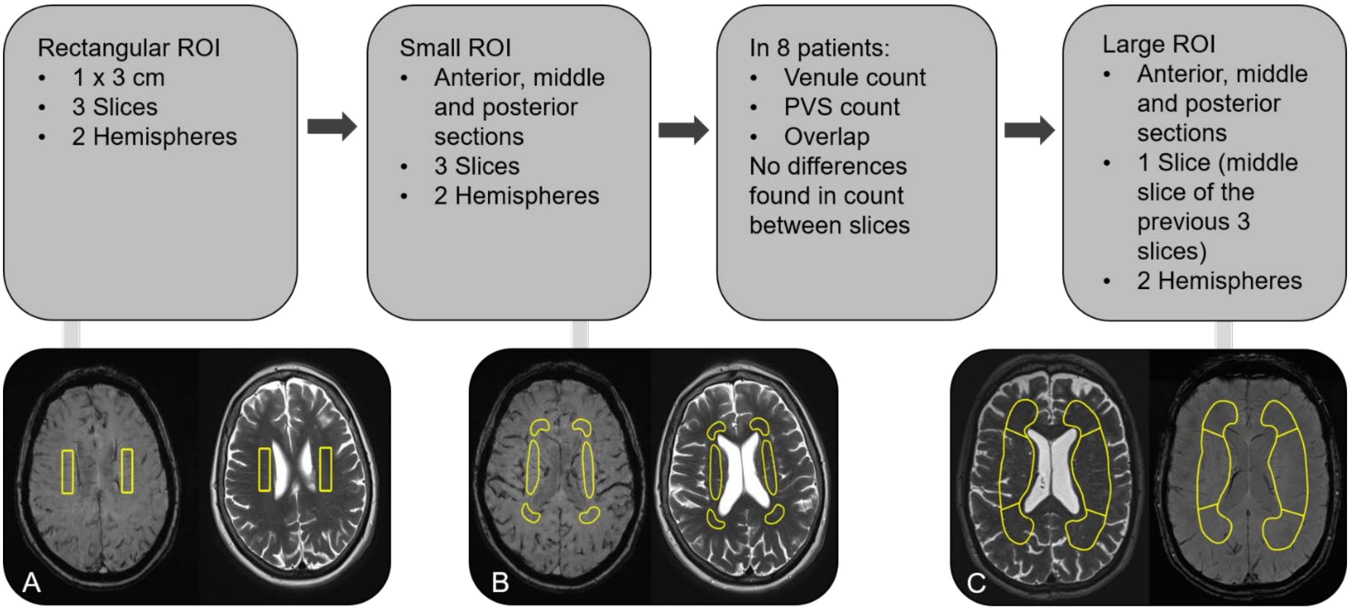

Figure I. Overview of the process of the development of the region of interest (ROI). A) First ROI, we would miss out on venules and PVS. B) The small ROI contained anterior, middle and posterior sections. This was applied in 8 patients. With this ROI we would still miss out on venules and perivascular spaces (PVS) further away from the ventricles. Thus we enlarged the ROI. C) We applied a larger ROI in only 1 slice since the counts and overlap were similar between the slices and to decrease time to count.

MRI and retinal imaging

iSVD study: We performed MRI using a 3T Siemens Magnetom Verio scanner (Siemens Healthcare, Erlangen, Germany) and a 12-channel head coil. We acquired axial 2D T2-weighted, axial 3D GRE, and 2D fluid-attenuated inversion recovery (FLAIR) sequences[1].

MSS-3: We used a 3T Siemens Magnetom Prisma MRI scanner with a 32-channel head coil (Siemens Healthcare, Erlangen, Germany) to acquire isotropic axial 3D T2-weighted SPACE, sagittal 3D T1-weighted MPRAGE, isotropic sagittal 3D FLAIR SPACE and axial 3D SWI. We performed phase contrast MRI to measure pulsatility in the superior sagittal sinus (SSS), straight sinus (StS) and transverse sinuses (TS) as described previously[2]. We used a 2D cine phase-contrast pulse sequence with retrospective peripheral pulse gating to obtain 32 velocity images per cardiac cycle. To measure venous flow waveforms, we selected a coronal-oblique slice that intersected the SSS at 2 cm above the torcular and through the midpoint of the StS. Retinal images were acquired with a SPECTRALIS imaging platform (Heidelberg Engineering, Heidelberg, Germany). We employed a circular scan around the optic nerve head, generating 30° infrared (IR) images (8.8 x 8.8 mm) of the fundus via a scanning laser ophthalmoscope.

Image processing and analysis

Quantification of venules

To assess venules we used GRE and SWI sequences. For the iSVD study, we had pre- and post-contrast GRE scans. The first MRI scan was immediately followed by intravenous infusion of an ultrasmall superparamagnetic particles of iron oxide (USPIO) contrast agent (ferumoxytol). Eight of the twelve patients had an additional scan immediately after infusion. All twelve patients were scanned at 24-30h and at four weeks after infusion[1]. The post-contrast GRE made it more complicated to differentiate between venules and arterioles as the contrast agent might highlight arterioles and venules while vessels visible on pre-contrast GRE are most likely to be venules. Therefore we only used the pre-contrast GRE (from now on mentioned as GRE), however the post-contrast GRE scans were still available for comparison if needed.

All sequences we used were uploaded into Carestream PACS software version 11.3.2.0220 (Carestream Health, Inc., 2011). Most sequences were already lined up. If images were not lined up this was done manually by using linking and referencing tool available in Carestream. We also used drawing tools available in Carestream to draw the ROIs.

We considered hypointense vessel-like structures on the GRE and SWI sequences to be venular. First we applied the ROI and adjusted it to the scan. Then we counted all visible venules per section, i.e. anterior, middle or posterior, within the ROI in both hemispheres. Combining all the counts in both hemispheres (six sections in total) results in a total venular count per slice. If a venule was visible in two sections, e.g. middle and posterior, venule we included the count of the venule in the section where it originated from. In order to avoid double counting of venules, adjacent slices were reviewed. Maximum intensity projections were available for MSS-3. These were used to verify the presence of a venule when the

venule was not very clearly visible on the SWI. However, venules were only counted when they were visible on the SWI.

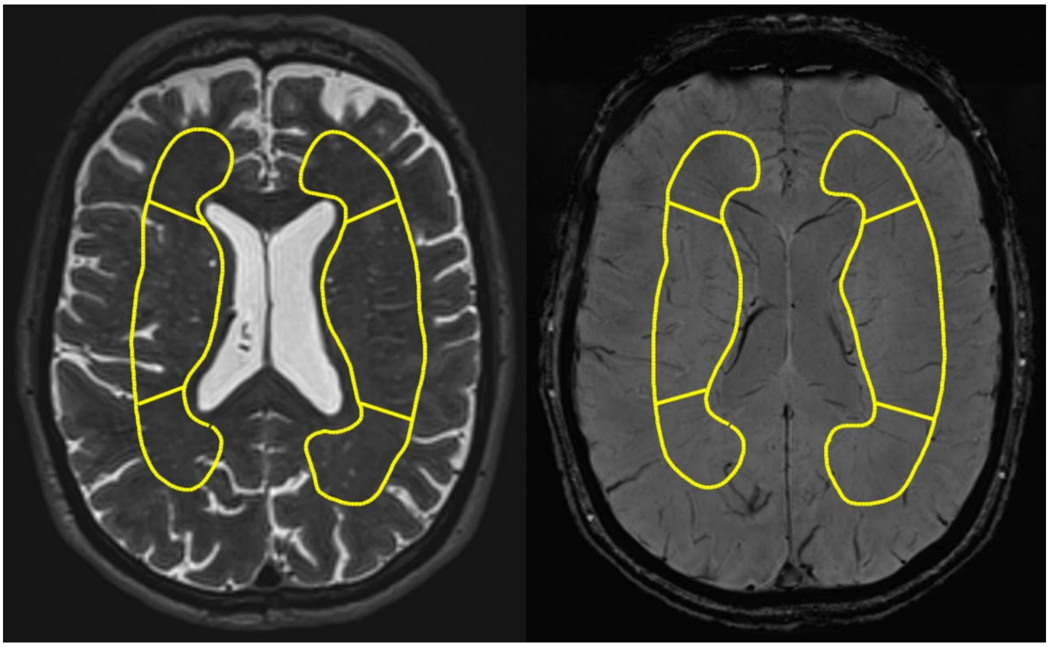

Figure II. An example of the final ROI in T2-weighted image (left) and susceptibility weighted imaging (right).

Quantification of PVS

After application of the ROI we counted PVS using T2-weighted images. In the CSO, PVS are seen as rounded or linear hyperintensities depending on direction of imaging slice[3]. PVS were counted in a similar way to venules, per part of ROI and per hemisphere. Combining these counts results in a total CSO PVS count per slice, calculated similar to the above for venules. Similar to venules, to avoid double counting of linear PVS, adjacent slices were reviewed. Since the ROI might overlap sulci in some patients, reviewing adjacent slices also prevents mistaking CSF in sulci as PVS as they might look similar.

Assessment of overlap

First we explored the use of computationally co-registered images using various in-house and open source software, e.g. FSL (https://fsl.fmrib.ox.ac.uk/fsl/fslwiki) and SPM (https://www.fil.ion.ucl.ac.uk/spm/), to improve the assessment of overlap. GRE and SWI were registered to the T2-weighted images. However, the registration process resulted in loss of image resolution which reduced the visibility of venules. Thus we compared the GRE and SWI images with the T2-weighted images to assess overlap. We determined the location of overlap based on a relating point in Carestream in both scans and by comparing the surrounding structures. The T2-weighted images from MSS-3 were resampled to create a similar slice thickness to SWI. We used this as an extra confirmation of PVS, but overlap was based on the original images. Additionally, we used sagittal T1-weighted images for MSS-3 to trace PVS and confirm overlap. We considered venules and PVS to overlap when they had the same location, shape and direction. However, determining overlap proved to be difficult due to reduced visibility of venules and PVS due to WMHs, stroke lesions or movement artefacts. When venules and PVS were less visible it was harder to determine if they had the same location, shape and direction. We created categories of overlap to describe cases where

the venule and PVS did seem to overlap despite not having the exactly same shape and direction (see Table I).

Some venules and PVS seemed to only partially overlap, e.g. a PVS could overlap with a venule based on same location, shape and direction, but only for a part of the venule. This overlap was still scored as definite, probable, or possible overlap with the addition 'partial', e.g. partial possible overlap. When overlap could still not be confidently identified based on the categories it was not counted as overlap.

Table I. Overlap categories and partial overlap

Definite overlap	Same location, shape and direction. Both venule and PVS are (relatively) distinct and overlap is clearly seen
Probable overlap	Same location, similar shape and direction. Venule and/or PVS are (relatively) distinct, but less distinct than with definite overlap and overlap is less clearly seen
Possible overlap	Same location. Mostly same shape and direction. Venule and PVS (relatively) indistinct, possibly due to e.g. scan quality and/or overlapping WMH
Additional: Partial overlap	<50% overlap of venule and PVS

PVS; Perivascular space; WMH: White matter hyperintensity

Pulsatility measures: Using phase contrast images, a trained analyst, blinded to clinical, venular and PVS data, manually drew ROIs around the SSS, StS and left and right TS and in adjacent non-vascular background tissue. We subtracted the average background velocity from those of the vessel ROIs, corrected the background phase error and calculated the pulsatility index (PI) as ($Flow_{maximum}$ - $Flow_{minimum}$)/$Flow_{mean}$[2].

Retinal vessel analysis: The IR fundus images were processed with the Vascular Assessment and Measurement Platform for Images of the REtina (VAMPIRE; Web version, Universities of Edinburgh and Dundee), a validated software application for semi-automatic quantification of retinal vessel properties[4]. We analyzed the central arterial equivalent (CRAE), central venular equivalent (CRVE) and arteriole-to-venule ratio (AVR), representing the widths of the arterioles and venules, and the ratio of vessels widths.

SVD lesions: Brain SVD lesions were scored (ACCJ) and checked by an experienced neuroradiologist (JMW) blind to clinical data, venule scores, venular-PVS overlap, pulsatility and retinal results. We rated periventricular and deep WMH using the Fazekas scale[5] (each score range 0-3, and a combined Fazekas score, 0-6). PVS were scored in the BG and CSO on a validated scale (range 0-4)[3]. Lesions were defined according to the STRIVE criteria[6].

Inter-observer reliability analysis

To recreate a practical scenario, two observers with different levels of experience and training visually assessed the perivascular spaces (PVS) and the hypointense vessels in two different visual platforms and following similar, but not identical procedures (Table II) in the 12 datasets from the Inflammation in Small Vessel Disease (iSVD) study. The assessments were done blind to each-other's results. To evaluate the validity of the results presented and limits of agreement, inter-observer reliability was analyzed using Bland-Altman plots.

Table II. Details of the visual assessment carried-out for the inter-observer reliability analysis

	Observer 1 (ACCJ) – reference assessment	Observer 2 (MVH)
Image used in the assessment of the PVS	Raw axial T2-weighted (voxel dimensions 0.625mm x 0.625mm x 3mm, matrix 384 x 384 x 48)	
Image used in the assessment of suspected venules	Raw T2*-weighted gradient echo (voxel dimensions 0.75mm x 0.75mm x 0.75mm, matrix 320 x 320 x 48) and raw T2* magnitude multi-echo (voxel dimensions 0.9375mm x 0.9375mm x 2mm, matrix 256 x 256 x 72)	Raw T2*-weighted gradient echo (voxel dimensions 0.75mm x 0.75mm x 0.75mm, matrix 320 x 320 x 48)
Regions of Interest (ROI)	Anterior, middle and posterior sections in one axial slice in the centrum semiovale (note both observers assessed the same slice in each dataset)	
Regions visually assessed	Selected slice and adjacent slices	Only selected slice
Pre-processing	Manual alignment of the sequences used through the Link and Reference Tool in Carestream	None
Viewing platform	Carestream PACS software version 11.3.2.0220 (Carestream Health, Inc., 2011)	MRIcron version 12 12 2012
Assessment results	Visual count of PVS, suspected venules and their overlap in each section and cerebral hemisphere, and whether the perceived overlap was partial, probable or definite.	Visual count of PVS, suspected venules and their overlap in each section and cerebral hemisphere.

There was excellent inter-observer agreement in the number of PVS counted in the anterior ROI section (6 PVS in the left hemisphere (LH) and 4 in the right (RH), mean difference 0, standard deviation (SD) of the difference between observers 2 in LH and 1 in RH). However, Observer 2 generally overestimated the number of PVS in the middle and posterior sections of the ROI in both hemispheres (mean difference per section 2 or 3 PVS, SD 2). The agreement was very good to excellent in the visual counting of suspected venules and almost perfect in reporting the number of PVS and suspected venules that overlapped in all sections (Table III, Figure III).

Table III. Results of the inter-observer agreement analysis

	ROI section	Mean difference (95%CI) (visual counts, rounded to the nearest integer)
PVS	Anterior section LH	0 (-4, 4)
	Middle section LH	-3 (-7, 1)
	Posterior section LH	-3 (-8, 2)
	Anterior section RH	0 (-2, 2)
	Middle section RH	-2 (-7, 3)
	Posterior section RH	-2 (-7, 3)
Suspected venules	Anterior section LH	0 (-1, 1)
	Middle section LH	1 (-3, 5)
	Posterior section LH	0 (-4, 4)
	Anterior section RH	0 (-1, 1)
	Middle section RH	0 (-2, 2)
	Posterior section RH	0 (-1, 1)
Overlap	Anterior section LH	0 (-1, 1)
	Middle section LH	0 (-1, 1)
	Posterior section LH	0 (-1, 1)
	Anterior section RH	0 (0, 0)
	Middle section RH	0 (-1, 1)
	Posterior section RH	0 (-1, 1)

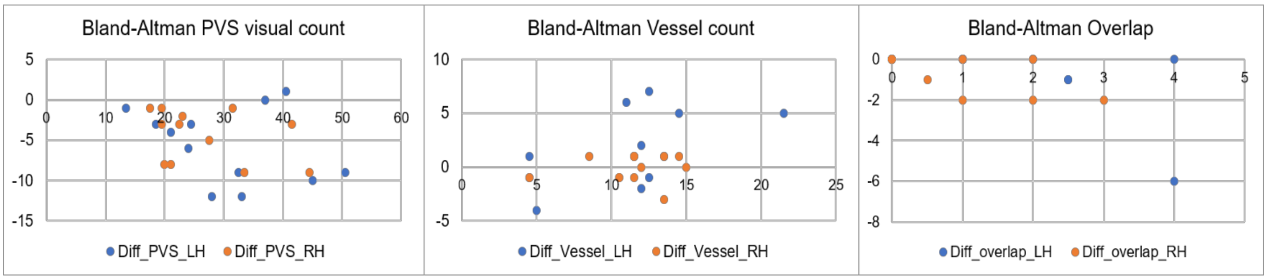

Figure III. Bland-Altman graphs of the inter observer agreement in the total number of counted PVS, suspected venules and overlap between the two. In the vertical axes are the differences in the counted features and in the horizontal axes the average of the counts by the two observers. The visual assessments for the left hemisphere are in blue and for the right hemisphere they are in orange.

In summary, despite inter-observer differences in the counts of PVS, the assessment of the overlap between suspected venules and PVS from both observers was almost identical.

Supplemental Tables

Table IV. Overview of venular visualization and quantification methods, ordered by field strength and year of publishing

Study	Field strength	MRI sequence	Subjects N, disease	ROI	Venular measurements	Main findings
Tan et al.,[7]	1.5T	GRE	17 MS subjects	Cerebral white matter	Visual: Presence of central vein sign in MS lesions	GRE can be used for in vivo visualization of perivenous location of MS lesions
Zamora et al.,[8]	1.5T and 3T	SWI	21 Neurosarcoidosis subjects	Periventricular CSO	Visual: Presence of deep medullary vein engorgement	DMV engorgement is a common finding in neurosarcoidosis patients
Ge et al.,[9*]	3T	SWI	13 MS subjects	Periventricular CSO	Computational: Segmentation of veins and quantification of venular voxels	Periventricular WM veins show reduced visibility in patients compared to controls
Horie et al.,[10†]	3T	SWI	33 MMD subjects	Periventricular CSO	Visual: Number of conspicuous DMVs (SWI stage)	SWI stage correlates strongly with ischemic presentations in MMD, cerebral blood flow and cerebrovascular reserve
Yan et al.,[11]	3T	SWI	158 Leukoaraiosis subjects	Periventricular CSO	Computational: Venous blood voxel count	Increased voxel count of DMVs was independently associated with both total WMH volume and regional WMH volume
Zhou et al.,[12*]	3T	SWI	15 RHRSP rats	Cerebral white matter	Visual: Prominence of veins using a grading scale	Increased visibility of veins and venous collagenosis are consequences of long-term hypertension in hypertensive rats
Mistry et al.,[13]	3T	GRE	13 MS subjects (Tested in 10 MS subjects[‖])	Cerebral white matter	Visual: Presence of a central vein in each WML	GRE allows differentiation between perivenous MS lesions and microangiopathic lesions

Yu et al.,[14†]	3T	SWI	43 Subacute ischemic stroke subjects	Periventricular CSO	Visual: Presence of prominent MVs	Prominent ipsilateral MVs was a significantly predict poor clinical outcome after subacute stroke and was associated with hypoperfusion
Campion et al.,[15]	3T	T2*-weighted and FLAIR	25 MS subjects	Periventricular CSO	Computational: Venous blood voxel count	FLAIR* allows the detection of veins in WML, differentiating between MS and SVD
Zhang et al.,[16]	3T	SWI	268 WMH subjects	Frontal, parietal and occipital regions in periventricular CSO	Visual: Four point grading score (0-3; continuous – not continuous) based on visibility and continuity of DMVs	Increased DMV score is associated with higher WMH volume and DMV scores were correlated with periventricular hyperintensity volume
Tallantyre et al.,[17]	3T and 7T	T2*-weighted and GRE	7 MS subjects	Cerebral white matter	Visual: Presence of central vein in WMH	7T is favorable in visualizing central veins in WML compared to 3T
Tallantyre et al.,[18]	7T	GRE	30 MS subjects 17 Non-MS subjects with WML	Cerebral white matter	Visual: Presence of central vein in WMH	>40% of WML in patients with MS were perivenous, <40% of WML in patients without clinical MS were perivenous
Sinnecker et al.,[19‡]	7T	T2*-weighted FLASH	38 MS subjects	1 x 4 cm in periventricular CSO	Visual: Number of veins, veins per ROI (number of veins divided by number of ROI) Computational: Periventricular vascular area[#]	Decreased venous density in MS patients. Venous density inversely correlated with periventricular and whole-brain T2 lesion count

Bouvy et al.,[20]	7T	TOF	5 Young healthy subjects<br>5 Older healthy subjects	BG, CSO, cortical GM	Visual: Presence of vein and correspondence to PVS	PVS correlated spatially with arteries, but not with veins
De Guio et al.,[21]	7T	GRE	13 CADASIL subjects	CSO	Computational: Total vein density (total of number of veins divided by total length of two segments), vein density in and outside WMH, length	Venous density is decreased within WMH and normal appearing WM in CADASIL patients compared to controls
Novelli et al.,[22‡]	7T	SWI	11 Sickle cell anemia subjects	1 x 4 cm ROI in periventricular CSO	Visual: Count, density of total count, long (9-10 mm) and short (3-5 mm) venules	Patients had a lower density of long venules and greater density of short venules. This was inversely related to memory performance and hemoglobin
Kuijf et al.,[23 §]	7T	GRE	24 Healthy subjects (Tested in 5 subjects[**])	Periventricular CSO; 5mm expanded ventricular surface	Computational: Count and density maps	Method allows fast and computational visualization and quantification of DMVs using 7T
Bouvy et al.,[24§]	7T	T2*-weighted	17 eAD subjects<br>12 aMCI subjects	Periventricular CSO	Computational: Number, density, tortuosity of veins	Number and density of DMVs do not differ. Tortuosity was higher in eAD and aMCI than controls

*Similar method; †similar method; ‡same ROI; §same method; ||Different subjects, not included in sample size; #Voxels of periventricular venules within bilateral ROI with intensity lower than 10% of the mean intensity of surrounding WMH. Periventricular area (mm^2); **Different subjects, not included in sample size; aMCI: Amnestic mild cognitive impairment; BG: Basal ganglia; CADASIL: Cerebral autosomal-dominant arteriopathy with subcortical infarcts and leukoencephalopathy; CSO: Centrum semiovale; DMV: Deep medullary veins; eAD: Early Alzheimer's disease; FLAIR: Fluid attenuation inversion recovery; FLASH: Fast low angle shot; GM: Grey matter; GRE: Gradient echo; MS: Multiple sclerosis; MMD: Moyamoya disease; MV: Medullary vein; RHRSP: Stroke-prone renovascular hypertensive rat; ROI: Region of interest; SWI: Susceptibility weighted images; TOF: Time of flight; WM: White matter; WMH: White matter hyperintensity; WML: White matter lesion

Table V. MRI parameters

Scan	TR (ms)	TE (ms)	FA (°)	Acquired voxel size (mm)	Slices (N)	Acquisition time (min:sec)	Other
iSVD study							
Axial 2D PROPELLER T2-weighted	11400	120	90	0.63 x 0.63 x 3	48	4:24	
Axial 3D GRE	40	20	15	0.75 x 0.94 x 3	48	5:35	
Axial 2D PROPELLER FLAIR	9100	125	130	0.94 x 0.94 x 3	48	4:53	
MSS-3							
Sagittal T1-weigted MPRAGE	2500	4.37	7	1 x 1 x 1	192	3:45	TI= 1100 ms
Transverse T2-weighted	3200	408	-	0.94 x 0.94 x 0.90	176	3:42	
SWI	28	20	9	0.63 x 0.63 x 3	52	4:02	
Sagittal 3D FLAIR	5000	388	-	1 x 1 x 1	192	5:57	TI=1800 ms
Venous sinuses flow (coronal-oblique)	21.70	6.59	12	0.71 x 0.71 x 5	1	~2:00	1 average

FA: Flip angle; FLAIR: Fluid-attenuated inversion recovery; GRE: Gradient echo; TE: Echo time; TI: Inversion time; TR: Repetition time

Table VI. Association venular count with demographic characteristics and vascular risk factors

	β	95% CI
Stroke subtype	0.106	-0.188, 0.400
Hypertension	0.128	-0.153, 0.409
hyperlipidemia	0.001	-0.301, 0.304
History of smoking	-0.114	-0.395, 0.167
Diabetes	-0.256	-0.531, 0.020
Vascular risk factors (0-4)*	-0.055	-0.343, 0.232
Diastolic BP	0.269	-0.071, 0.609
Salt use yes/no $	-0.049	-0.316, 0.216

Multivariable linear regression analyses, adjusted for age, sex and systolic blood pressure. *Composite score of presence of hypertension, hyperlipidemia, history of smoking and diabetes (0-4); $Use of salt in diet yes or no BP: Blood pressure; CI: Confidence interval

Table VII. Association venular count with retinal vasculature

	β	95% CI
AVR	0.004	-0.280, 0.289
CRVE	0.022	0.154, 0.628
CRAE	0.015	-0.282, 0.313

Multivariable linear regression analyses, adjusted for age, sex and systolic blood pressure. AVR: Arteriole-to-venule ratio; BP: Blood pressure; CI: Confidence interval; CRAE: Central Retinal Arterial Equivalent (arterial width); CRVE: Central Retinal Venular Equivalent (venular width)

Supplemental Figures

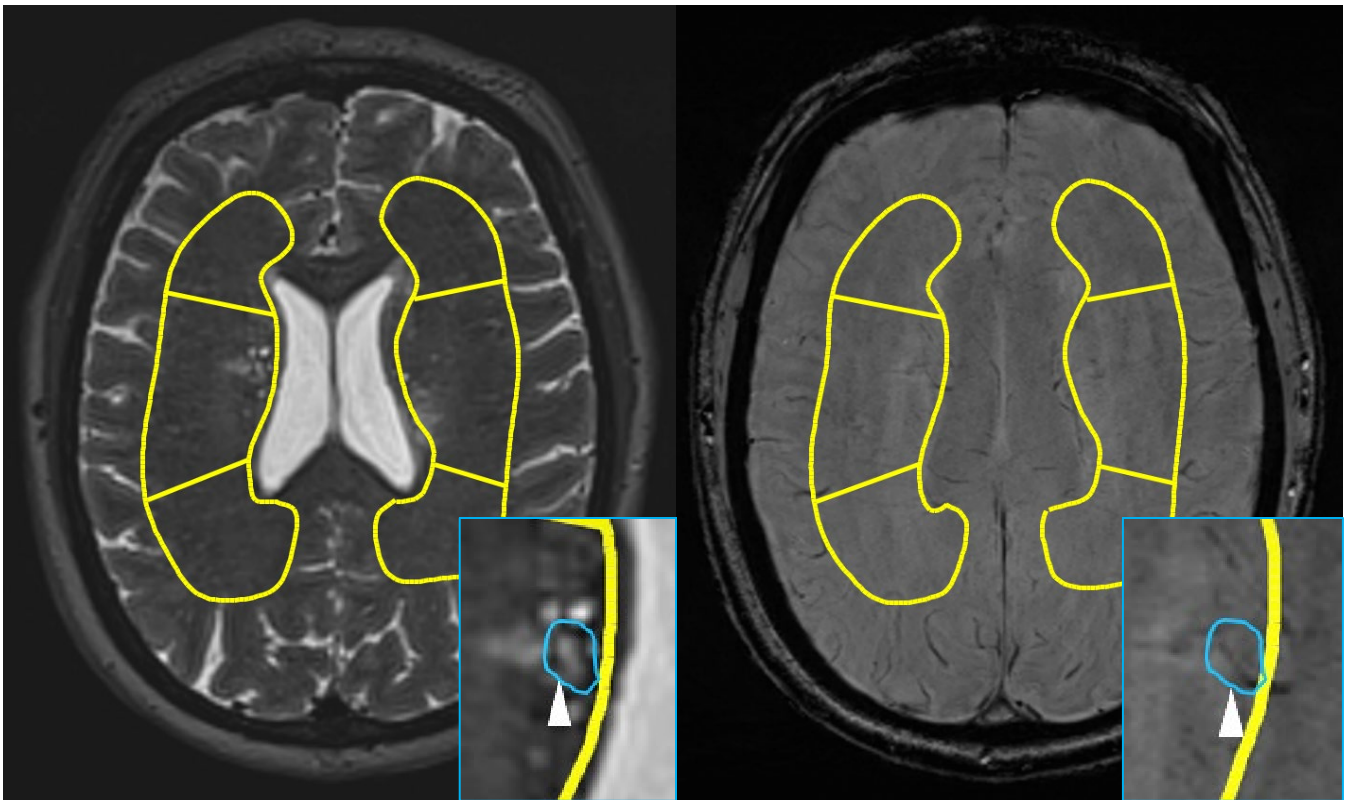

Figure IV. PVS (left inlay) at the ventricular surface where it overlaps with a venule (right inlay).

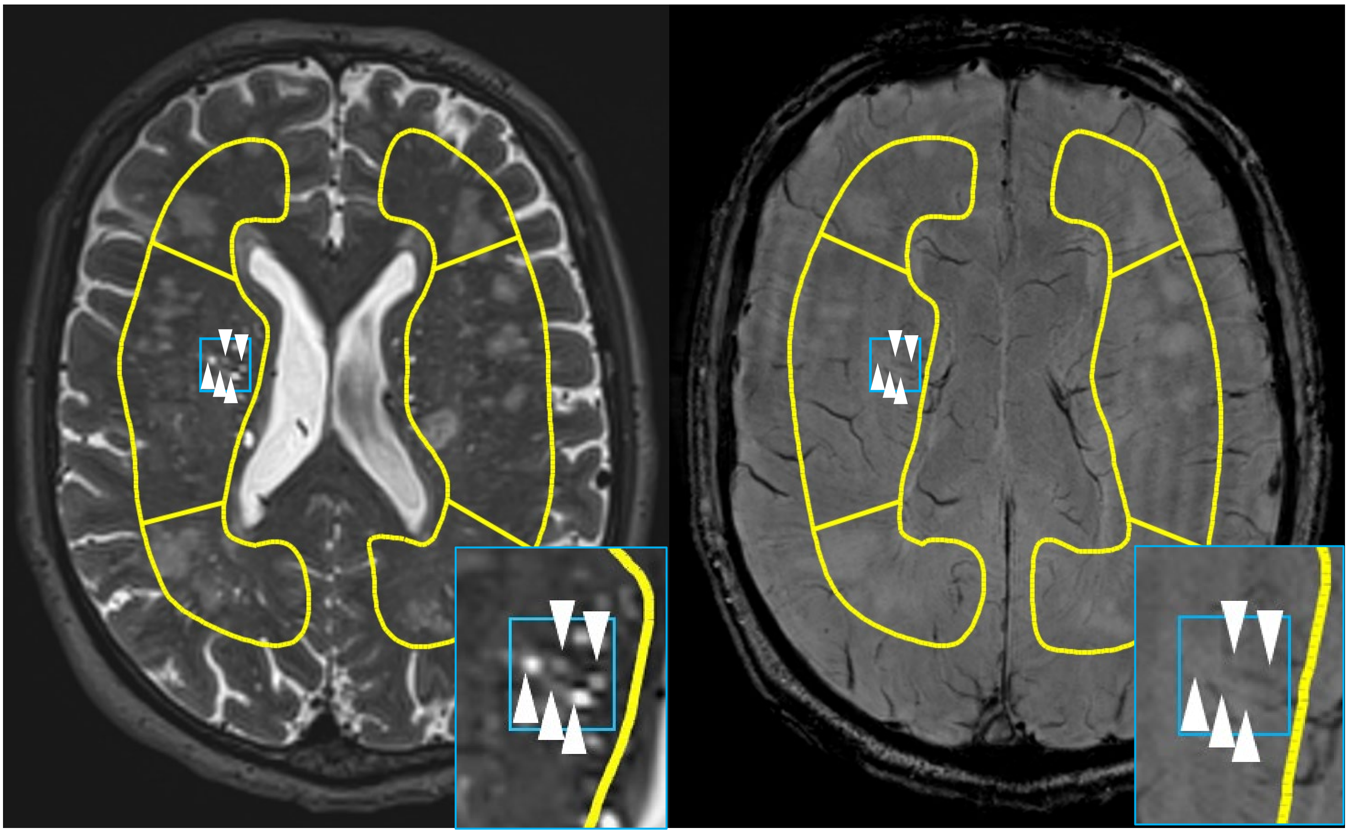

Figure V. Two venules (right) with overlapping PVS. PVS have a different shape and seem to be more punctate. However, the most posterior PVS seems to consist of two rounded hyperintensities and a linear in between.

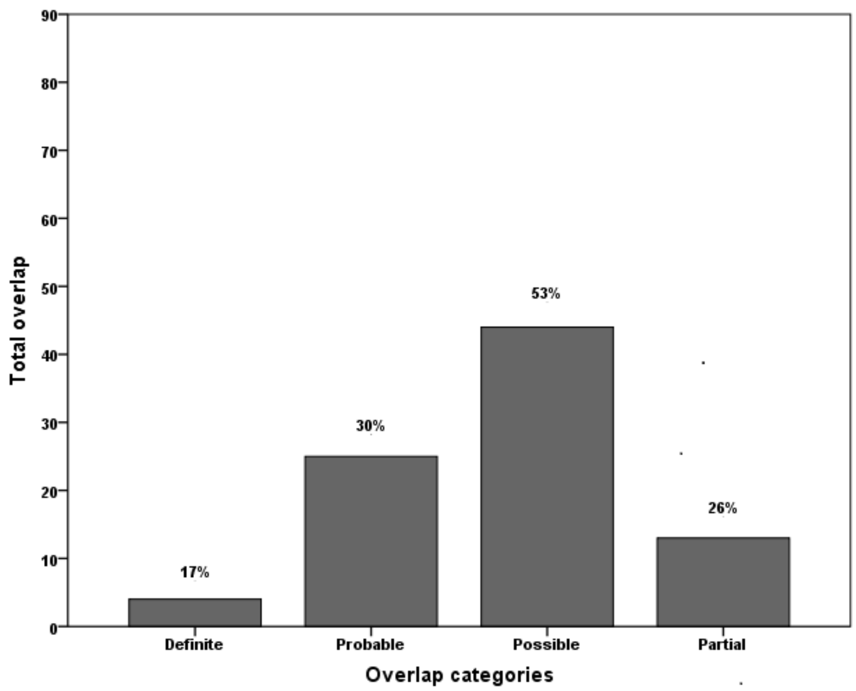

Figure VI. Overview of overlap categories in the total sum of overlap. Possible overlap occurred the most (53% of the venules with overlap). Additional partial overlap was found in 26% of overlap.

References

1. Thrippleton MJ, Blair GW, Valdes-Hernandez MC, Glatz A, Semple SI, Doubal F, et al. MRI Relaxometry for Quantitative Analysis of USPIO Uptake in Cerebral Small Vessel Disease. *International Journal of Molecular Sciences.* 2019;20:776.
2. Blair GW, Thrippleton MJ, Shi Y, Hamilton I, Stringer M, Chappell F, et al. Intracranial functional haemodynamic relationships in patients with cerebral small vessel disease. *bioRxiv.* 2019:572818.
3. Potter GM, Chappell FM, Morris Z, Wardlaw JM. Cerebral perivascular spaces visible on magnetic resonance imaging: development of a qualitative rating scale and its observer reliability. *Cerebrovascular Diseases.* 2015;39:224-231.
4. Trucco E, Ballerini L, Relan D, Giachetti A, MacGillivray T, Zutis K, et al. Novel VAMPIRE algorithms for quantitative analysis of the retinal vasculature. Paper presented at: 2013 ISSNIP Biosignals and Biorobotics Conference: Biosignals and Robotics for Better and Safer Living (BRC)2013.
5. Fazekas F, Kleinert R, Offenbacher H, Schmidt R, Kleinert G, Payer F, et al. Pathologic correlates of incidental MRI white matter signal hyperintensities. *Neurology.* 1993;43:1683-1683.
6. Wardlaw JM, Smith EE, Biessels GJ, Cordonnier C, Fazekas F, Frayne R, et al. Neuroimaging standards for research into small vessel disease and its contribution to ageing and neurodegeneration. *The Lancet Neurology.* 2013;12:822-838.
7. Tan IL, Van Schijndel RA, Pouwels PJ, Van Walderveen MA, Reichenbach JR, Manoliu RA, et al. MR venography of multiple sclerosis. *American Journal of Neuroradiology.* 2000;21:1039-1042.
8. Zamora C, Hung S-C, Tomingas C, Atkinson C, Castillo M. Engorgement of deep medullary veins in neurosarcoidosis: a common-yet-underrecognized cerebrovascular finding on SWI. *American Journal of Neuroradiology.* 2018;39:2045-2050.
9. Ge Y, Zohrabian VM, Osa EO, Xu J, Jaggi H, Herbert J, et al. Diminished visibility of cerebral venous vasculature in multiple sclerosis by susceptibility-weighted imaging at 3.0 Tesla. *Journal of Magnetic Resonance Imaging.* 2009;29:1190-1194.
10. Horie N, Morikawa M, Nozaki A, Hayashi K, Suyama K, Nagata I. "Brush sign" on susceptibility-weighted MR imaging indicates the severity of moyamoya disease. *American Journal of Neuroradiology.* 2011;32:1697-1702.
11. Yan S, Wan J, Zhang X, Tong L, Zhao S, Sun J, et al. Increased visibility of deep medullary veins in leukoaraiosis: a 3-T MRI study. *Frontiers in Aging Neuroscience.* 2014;6:144.
12. Zhou M, Mao L, Wang Y, Wang Q, Yang Z, Li S, et al. Morphologic changes of cerebral veins in hypertensive rats: venous collagenosis is associated with hypertension. *Journal of Stroke and Cerebrovascular Diseases.* 2015;24:530-536.
13. Mistry N, Abdel-Fahim R, Samaraweera A, Mougin O, Tallantyre E, Tench C, et al. Imaging central veins in brain lesions with 3-T T2*-weighted magnetic resonance imaging differentiates multiple sclerosis from microangiopathic brain lesions. *Multiple Sclerosis Journal.* 2016;22:1289-1296.
14. Yu X, Yuan L, Jackson A, Sun J, Huang P, Xu X, et al. Prominence of medullary veins on susceptibility-weighted images provides prognostic information in patients with subacute stroke. *American Journal of Neuroradiology.* 2016;37:423-429.
15. Campion T, Smith R, Altmann D, Brito G, Turner B, Evanson J, et al. FLAIR* to visualize veins in white matter lesions: A new tool for the diagnosis of multiple sclerosis? *European Radiology.* 2017;27:4257-4263.

16. Zhang R, Zhou Y, Yan S, Zhong G, Liu C, Jiaerken Y, et al. A Brain Region-Based Deep Medullary Veins Visual Score on Susceptibility Weighted Imaging. *Frontiers in Aging Neuroscience.* 2017;9:269.
17. Tallantyre EC, Morgan PS, Dixon JE, Al-Radaideh A, Brookes MJ, Evangelou N, et al. A comparison of 3T and 7T in the detection of small parenchymal veins within MS lesions. *Investigative Radiology.* 2009;44:491-494.
18. Tallantyre E, Dixon J, Donaldson I, Owens T, Morgan P, Morris P, et al. Ultra-high-field imaging distinguishes MS lesions from asymptomatic white matter lesions. *Neurology.* 2011;76:534-539.
19. Sinnecker T, Bozin I, Dörr J, Pfueller CF, Harms L, Niendorf T, et al. Periventricular venous density in multiple sclerosis is inversely associated with T2 lesion count: a 7 Tesla MRI study. *Multiple Sclerosis Journal.* 2013;19:316-325.
20. Bouvy WH, Biessels GJ, Kuijf HJ, Kappelle LJ, Luijten PR, Zwanenburg JJ. Visualization of perivascular spaces and perforating arteries with 7 T magnetic resonance imaging. *Investigative Radiology.* 2014;49:307-313.
21. De Guio F, Vignaud A, Ropele S, Duering M, Duchesnay E, Chabriat H, et al. Loss of venous integrity in cerebral small vessel disease: a 7-T MRI study in cerebral autosomal-dominant arteriopathy with subcortical infarcts and leukoencephalopathy (CADASIL). *Stroke.* 2014;45:2124-2126.
22. Novelli EM, Sarles CE, Aizenstein HJ, Ibrahim TS, Butters MA, Ritter AC, et al. Brain venular pattern by 7T MRI correlates with memory and haemoglobin in sickle cell anaemia. *Psychiatry Research: Neuroimaging.* 2015;233:18-22.
23. Kuijf HJ, Bouvy WH, Zwanenburg JJ, Schultz TBR, Viergever MA, Vincken KL, et al. Quantification of deep medullary veins at 7 T brain MRI. *European Radiology.* 2016;26:3412-3418.
24. Bouvy WH, Kuijf HJ, Zwanenburg JJ, Koek HL, Kappelle LJ, Luijten PR, et al. Abnormalities of cerebral deep medullary veins on 7 tesla MRI in amnestic mild cognitive impairment and early Alzheimer’s Disease: a pilot study. *Journal of Alzheimer's Disease.* 2017;57:705-710.
